# Supplementary material for: Subjective and objective effects of anxiety and fatigue on social function in patients with enterostomy and their family caregivers
Source: PLoS One. 2024 Nov 8;19(11):e0309991. doi: 10.1371/journal.pone.0309991 (PMC11548842; doi:10.1371/journal.pone.0309991)
Supplement: S1 Raw data — (ZIP) [file pone.0309991.s001.zip › Raw Data Description/Raw Data Description.docx]

Raw Data Description

①② is the original data of Table 1

③④ is the original data of Table 2: where "D" represents social function "S" represents anxiety "M" represents fatigue

⑤ is the original data of Tables 3, 4, 5, 6, 7 and Figure 3
